# Clinical Significance of Circulating miR-1273g-3p and miR-122-5p in Pancreatic Cancer

**DOI:** 10.3389/fonc.2020.00044

**Published:** 2020-02-04

**Authors:** Tommaso Mazza, Domenica Gioffreda, Andrea Fontana, Tommaso Biagini, Massimo Carella, Orazio Palumbo, Evaristo Maiello, Francesca Bazzocchi, Angelo Andriulli, Francesca Tavano

**Affiliations:** ^1^Laboratory of Bioinformatics, Fondazione IRCCS Casa Sollievo della Sofferenza, Foggia, Italy; ^2^Division of Gastroenterology and Research Laboratory, Fondazione IRCCS Casa Sollievo della, Foggia, Italy; ^3^Unit of Biostatistics, Fondazione IRCCS Casa Sollievo della Sofferenza, Foggia, Italy; ^4^Division of Medical Genetics, Fondazione IRCCS Casa Sollievo della Sofferenza, Foggia, Italy; ^5^Department of Oncology, Fondazione IRCCS Casa Sollievo della Sofferenza, Foggia, Italy; ^6^Department of Surgery, Fondazione IRCCS Casa Sollievo della Sofferenza, Foggia, Italy

**Keywords:** pancreatic cancer, circulating microRNA, expression profile, diagnostic performance, prognosis

## Abstract

The burden of pancreatic cancer (PanC) requires innovation in the current diagnostic approach. This study aimed to uncover new circulating microRNAs (miRNAs) that would distinguish patients with PanC from healthy subjects (HS) compared with the cancer antigen 19-9 (CA 19-9), and predict patients' clinical phenotypes and outcomes. MiRNA expression profiles in plasma were investigated by using a two-stage process. In a discovery phase, miRNAs levels were analyzed using the GeneChip™ miRNA 4.0 Affymetrix assay in 10 pools of plasma samples from PanC patients and HS; in a validation phase, significantly altered miRNAs were re-tested in independent cohorts of cancer patients and controls by droplet digital PCR (ddPCR). The diagnostic performance of the resulting miRNAs was compared to CA 19-9 determinations, and the associations of miRNAs plasma levels with patients' clinical phenotypes and outcomes were also taken into account. Bioinformatics selection of miRNAs differentially expressed in plasma uncovered miR-18a-5p, miR-122-5p, miR-1273g-3p, and miR-6126 as candidate oncogenic miRNAs in PanC. The ddPCR technology confirmed the significant over-expression of miR-122-5p, miR-1273g-3p, and miR-6126 in PanC compared to HS, in line with the trend of the CA 19-9 levels. Plasma levels of miR-1273g-3p, in combination with CA 19-9, showed higher power in distinguishing PanC patients from HS compared to the CA 19-9 tested alone, with a gain in both sensitivity and negative predictive value indicating a low false-negative rate (SE = 90.2% and NPV = 92.3% vs. SE = 82.1% and NPV = 87.9%). None of the oncogenic miRNAs were able to distinguish between a neoplastic and a proliferative/inflammatory disease of the pancreas, and were not able to stratify subjects according to the clinical risk for the disease. The only valuable association in PanC patients was found between miR-1273g-3p and tumor stage, and increased miR-122-5p levels emerged as independent negative prognostic factor for PanC patients (HR = 1.58, 95% CI = 1.03–2.43, *p* = 0.037). Our data highlighted a role for circulating miR-1273g-3p and miR-122-5p as new diagnostic and prognostic biomarkers for PanC.

## Introduction

Pancreatic cancer (PanC) is one of the most lethal forms of cancer (1). Although the early detection of the disease may benefit patients, a low incidence and limitations of current pancreatic imaging make it difficult to establish a screening program. Risk factors for developing PanC include both genetics and environmental factors. Specific mutations in multiple genes have been found in roughly 10% of PanC patients, with varying penetrance and degree of the cancer risk for each of them (2). Environmental risk factors include tobacco exposure, heavy alcohol intake, chronic pancreatitis, diet, obesity, cholecystectomy and/or gastric resection, Helicobacter Pylori infection, non-O blood groups, and type 1, 2, and 3 diabetes (3, 4). More in detail, the risk for diabetes-associated PanC has been reported to increase in patients with early-onset diabetes, and to decrease negatively along the years from the diabetes diagnosis (5, 6).

As miRNAs are resistant to degradation in body fluids, they may serve as biomarkers for human diseases, including PanC (7, 8). Expression levels of several miRNAs (miR-18a,-21,-155,-185,-196a,-210,-212, and -483) were reported to be increased in both tissue and serum/plasma from PanC patients, and were useful to discriminate adenocarcinoma from non-cancerous lesions of the gland (9–11). Similarly, increased expression levels of miR-20a,-21,-24,-99a,-185, and -191 in serum were able to differentiate PanC patients from either controls and subjects with pancreatitis (12). A prognostic role for circulating miRNAs levels in PanC has also been postulated as increased levels of miR-221 and −21 were reported to correlate with distal metastases and clinical outcomes, respectively (9, 13).

In addition, data are also available on the diagnostic efficiency of the concurrent evaluation of specific miRNA or miRNAs panels in combination with cancer antigen 19-9 (CA 19-9) determinations (14–17). Liu et al. by determining the relative abundances of seven miRNAs (miR-16,-21,-155,-181a,-181b,-196a, and -210) in plasma, and their diagnostic utility for PanC, found that the combination of miR-16,-196a, and CA 19-9 was more effective for discriminating patients with PanC from those with chronic and normal controls compared with the miRNA panel (miR-16,-196a) or CA19-9 alone (14). Furthermore, these authors found that miR-16 and -196a combined with CA19-9 were effective to identify PanC patients in stage 1. Two different miRNAs panels, including 4 (miR-145,-150,-223,-636) and 10 miRNAs (miR-26b,-34a,-122,-126^*^,-145,-150,-223,-505,-636,-885), respectively, were tested by Schultz NA and colleagues (15). The former panel combined with the CA 19-9 values, showed a better diagnostic performance compared with the CA19-9 alone; in addition, improved performance in diagnosing PanC patients in stage IA-IIB was observed for both miRNAs panels.

In a previous study (16), we used the droplet digital PCR (ddPCR) to validate the findings of Li et al. about miR-1290 as circulating biomarker for PanC (17). In agreement with the results of these authors, who used quantitative PCR to quantify miR-1290 plasma levels in patients with PanC compared to HS, we found a significant increase of absolute plasma levels of miR-1290 in PanC. However, in contrast to the findings of those authors, we observed that it was only after combining miR-1290 levels to those of the CA 19-9 that we could separate the cancer cohort from the HS cohort. In addition, the discriminating ability was higher when only PC patients with low or slightly increased CA 19-9 levels were compared with HS.

Herein, by using a two-stage process, we aimed to identify novel miRNAs in plasma useful to distinguish the PanC from HS compared with the CA19-9, and to predict the patients' clinical phenotypes and outcomes.

## Materials and Methods

### Patients and Controls

Between November 2011 and May 2016, 322 subjects were enrolled at the Divisions of Gastroenterology, Surgery, and Oncology of “Casa Sollievo della Sofferenza” Hospital, San Giovanni Rotondo (Italy) into the study, after signing an informed consent form. The study was approved by the local Ethics Committee (Prot. No. 96/CE/2011). Candidates in the study were healthy subjects (HS, no. = 170), and patients with PanC (no. = 132). In addition, patients harboring a proliferative or an inflammatory process within the pancreas, i.e., those with intraductal papillary neoplasm (IPMN, no. = 10) or chronic pancreatitis (no. = 10), were also considered in the validation part of the study and served as internal controls.

From either HS and the PanC cohort, the following information about environmental factors considered to contribute to PanC development were collected: age ≥55 years, body mass index >30, smoking, alcohol abuse, diabetes, and a family history of cancer. Based on the number of previous factors recalled by enrolled individuals, the two cohorts were classified as at low (i.e., 0–1 risk factors), intermediate (i.e., 2–3 features), and high (i.e., 4–6 factors) risk. In addition, from a small subgroup of individuals, it was possible to get information about the date of diagnosis of diabetes. Accordingly, if diabetes was ascertained within 2 years prior to enrollment into the study (for the HS cohort) or to the PanC diagnosis (for the cancer cohort), subjects were classified as early-onset diabetes; if diabetes was ascertained more than 2 years prior to enrollment into the study (for the HS cohort) or to the PanC diagnosis (for the cancer cohort), subjects were classified as late-onset diabetes.

As expected, the PanC cohort included older patients (median age at diagnosis: PanC 70 years vs. HS 62 years) and a higher prevalence of males (PanC 45.5% vs. HS 44.1%). In over two-thirds of patients, the cancer was located in the pancreatic head. At the pre-operative work-up, 23.5% presented with a resecitable disease, 32.6% with locally advanced cancer, and 43.9% with metastatic disease (18). Only 25 of the 31 patients with resectable disease underwent resective surgery, being judged fit for surgery. According to the American Joint Committee on Cancer (AJCC) tumor/node/metastasis (TNM) classification and staging system for PanC (19), the 46.8% of patients were in stage IV, 33.9% in stage III, and 19.3% in stage I-II. Adjuvant chemotherapy was administered to 44.6% of patients, with the remaining individuals considered ineligible for chemotherapy.

### Study Design

**Figure 1:**
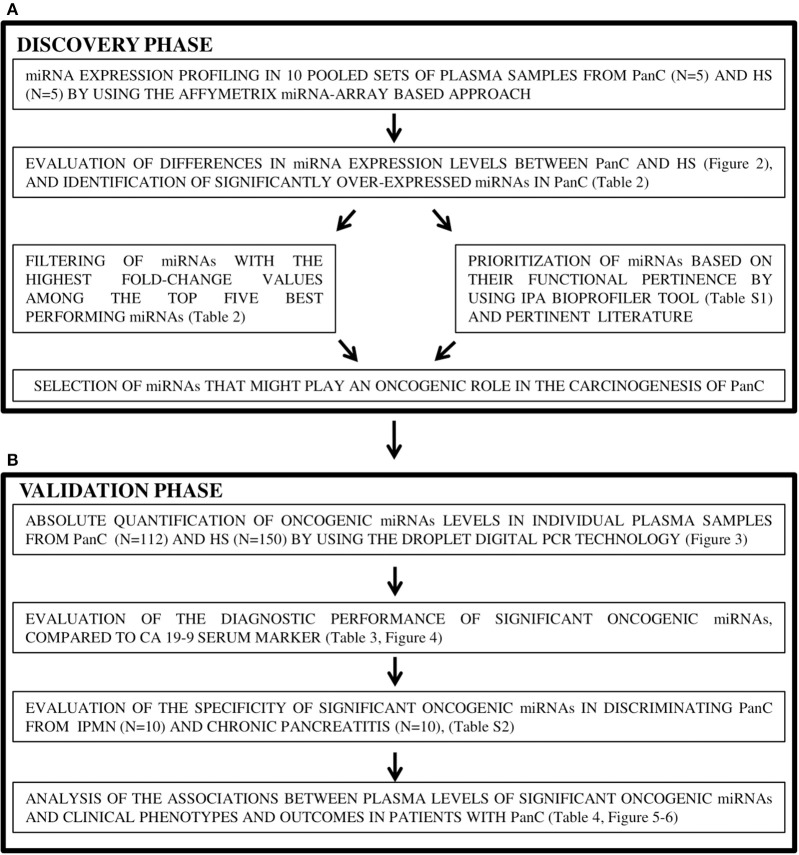
Study design of the two-stage process for the identification **(A)** and validation **(B)** of candidate oncogenic miRNAs in pancreatic cancer. PanC, pancreatic cancer; HS, Healthy subjects.

A flow chart of the study design is presented in Figure 1. From the cohort of HS, a few individuals were selected and evaluated in the discovery phase of the study, and the remaining majority were used for the validation test. Similarly, from the cohort of PanC, a few patients were used in the discovery phase, and the remaining majority in the validation phase.

In the discovery phase, circulating miRNAs levels were profiled in a total of 10 pools of plasma samples, 5 for the PanC and 5 for the HS subsets. Each pool was constituted from 4 HS with similar risk factors for PanC development; i.e., plasma samples from HS in the low-risk group were pooled in Pool_1_, plasma from those in the intermediate-risk formed the Pool_2_, and plasma from HS in the high-risk category were pooled in Pool_3_. In addition, plasma from 4 HS with early-onset diabetes served to form Pool_4_, and 4 HS with late-onset diabetes were included in Pool_5_. Five different plasma pools were also constituted for the PanC cohort: based on the preoperative stage of the disease, Pool_6_, Pool_7_, and Pool_8_ each included 4 samples from PanC with resectable, locally advanced and metastatic disease, respectively; in addition, two other pools were also formed, one for PanC patients with early- (Pool_9_), and one for those with late-onset diabetes (Pool_10_).

All remaining PanC patients (no. 112) and HS (no. 150), as well as those with proliferative (IPMN) or inflammatory (chronic pancreatitis) pancreatic conditions, were used in the validation phase, where the miRNAs discovered in pooled sets of samples were tested by means of the ddPCR system to confirm the results. For this validation cohort, demographic features, risk factors for the developing of PanC, and baseline clinical-pathological features of PanC are shown in Table 1. In this phase of the study, the diagnostic performance of the resulting miRNAs was compared to that of the CA 19-9 determination, and the associations of miRNAs plasma levels with patients' clinical phenotypes were also taken into account.

**Table 1:** Demographics characteristics, clinical risk factors in healthy subjects (HS) and patients with pancreatic cancer (PanC), and baseline clinical-pathological features of PanC patients included in the validation cohort.

	**PanC (*N* = 112)**	**HS (*N* = 150)**
Age, median (IQR)	70 (61–77.5)	62 (55–70)
<55 years, *N* (%)	13 (11.6)	36 (24)
≥55 years, *N* (%)	99 (88.4)	114 (76)
Gender, *N* (%)		
Male	52 (46.4)	68 (45.3)
Female	60 (53.6)	82 (54.7)
Risk score for PC Development, *N* (%)		
Low (0–1 risk factor)	13 (11.7)	23 (15.3)
Intermediate (2–3 risk factors)	83 (74.8)	117 (78)
High (> 4 risk factors)	15 (13.5)	10 (6.7)
Missing	1	
Body mass index, *N* (%)		
≤30	93 (85.3)	118 (78.7)
>30	16 (14.7)	32 (21.3)
Missing	3	
Smoker, *N* (%)		
No	51 (45.5)	71 (47.3)
Current/Past	61 (54.5)	79 (52.7)
Alcohol abuse (≥3 drinks a day), *N* (%)		
No	99 (88.4)	142 (94.7)
Yes	13 (11.6)	8 (5.3)
Diabetes, *N* (%)		
No	82 (73.2)	145 (96.7)
Yes	30 (26.8)	5 (3.3)
Family history of cancer, *N* (%)		
No	41 (38)	39 (26)
Yes	67 (62)	111 (74)
Missing	4	
Tumor location, *N* (%)		
Head	76 (67.86)	
Head/Body	3 (2.68)	
Body/Tail	33 (29.46)	
Pre-operative classification, *N* (%)		
Resectable	19 (17)	
Locally advanced	39 (35)	
Metastatic	54 (48)	
Surgery, *N* (%)		
No	98 (87.5)	
Yes	14 (12.5)	
Tumor stage, *N* (%)		
IB/IIA	3 (2.8)	
IIB	13 (12.3)	
III	36 (33.9)	
IV	54 (51)	
Missing	6	
Adjuvant therapy, *N* (%)		
No	46 (42)	
Yes	64 (58)	
Missing	2	

### Plasma Samples Collection and miRNAs Purification

From all studied patients and subjects, a blood sample was obtained at the completion of the diagnosis work-up, before surgery and/or administration of chemotherapy. Plasma samples were drawn in citrate tubes and centrifuged at 3,500 rpm for 10 min at room temperature within 1 h of withdrawal. Total RNA and miRNA fractions were isolated by using the miRNesy Serum/Plasma Kit (Cat. No. 217184, Qiagen, Hilden, Germany), according to the manufacturers' instructions. In details, two 200 μl of plasma aliquots were used for miRNAs purification in the discovery cohort. From each aliquot the RNA was eluted in 14 μl of RNase free water, and the elution's volumes from the two aliquots were combined to obtain a final volume of 28 μL. One 100 μl of plasma aliquot was processed for the validation assays. Furthermore, in order to increase the RNA yield, purified Torulla yeast RNA carrier (Cat. No. AM7120G, Ambion^®^, Thermo Fisher Scientific, Carlsbad, CA) at a concentration of 1 μg/ml of plasma was added to samples before the chloroform protocol step (20). Differences in recovery efficiency between different plasma samples and the effect of hemolysis on miRNAs content in plasma were limited, as previously reported (16). RNA purity and the presence of contaminants were evaluated by spectrophotometry, according to the A260/230 and A260/280 ratios, by using NanoDrop ND-2000 (Thermo Fisher Scientific, Carlsbad, CA). A fixed volume of the extracted RNA samples was used in the following miRNAs expression profiling and ddPCR experiments.

### miRNA Profiling

MiRNA expression profiling in plasma was performed by using GeneChip™ miRNA 4.0 Array (Affymetrix, Santa Clara, CA) which contains probe sets for 30,424 mature miRNAs from Sanger miRBase Release 20, and covers 203 organisms of all species including human, mouse, and rat. For each pool, 8 μl of total RNA was labeled using the 3 DNA Array Detection Flash Tag RNA Labeling Kit (http://www.genisphere.com), according to the manufacturer's instructions. Firstly, poly (A) tailing was carried out at 37°C for 15 min in a volume of 15 ml reaction mix that contained 1 ml Reaction Buffer, 1.5 ml MgCl_2_ (25 mM), 1 ml ATP Mix diluted 1:500 and 1 ml PAP enzyme. Subsequently, Flash Tag Ligation was performed at room temperature for 30 min by adding 4 ml of 5 Flash Tag Ligation Mix Biotin and 2 ml T4 DNA Ligase into 15 ml of reaction mix. Next, 2.5 ml of Stop Solution was added to stop the reaction. Each sample was hybridized on the array, washed, stained with the Affymetrix Fluidics Station 450 and scanned with the Affymetrix Gene Chip Scanner 3000 7G using the Command Console software (Affymetrix). The microarray data set was deposited in the Array Express database under the accession number E-MTAB-8378.

### Absolute Quantification of miRNA by Droplet Digital PCR

Quantitative measurements of miRNAs in plasma were carried out using a two-step droplet digital PCR (ddPCR), as previously reported (16). In the initial step, cDNA was synthesized from 1 μl of extracted RNA using the TaqMan miRNA Reverse Transcription Kit and miRNA-specific stem-loop primers (Thermo Fisher Scientific, Foster City, CA; cat. No. 4427975; hsa-miR-122-5p, Assay ID: 002245; hsa-miR-1273g-3pg-3p, Assay ID: 475626_mat; has-miR-6126, Assay ID: 475618_mat; hsa-miR-18a-5p, Assay ID: 002422) following manufacturer's instructions. Then, 5 μl of the synthesized cDNA was amplified by mixing 2X ddPCR Supermix for Probes (Bio-Rad Laboratories, Hercules, CA) and 20X TaqMan miRNA PCR primer probe set (Thermo Fisher Scientific, Carlsbad, CA), and quantified by the ddPCR system (Bio-Rad Laboratories, Hercules, CA).

### Statistical and Bioinformatics Analyses

Expression levels of circulating miRNAs and CA 19-9 were described as median along with interquartile range (IQR, i.e., first-third quartiles). Patients' characteristics were reported as median along with IQR or frequencies and percentages for continuous and categorical variables, respectively. Because of the skewed distribution of miRNAs levels (even after the log-transformation of data), comparisons between miRNAs levels and categorical variables (identifying patients' groups, risk categories or any other categorical clinical variable) were performed using Mann–Whitney *U* or Kruskal–Wallis tests, as appropriate. For each miRNA boxplots were produced, along with raw data points (log scale was used for the Y-axis).

The diagnostic performance of miRNAs and CA 19-9 levels in discriminating PanC patients from HS was assessed by the Receiver Operating Characteristic (ROC) curve analysis, with the estimation of the Area Under the ROC curve (AUC), along with its 95% confidence intervals (CI), computed with 2,000 stratified bootstrap replicates. Sensitivity (SE), specificity (SP), positive predictive value (PPV) and negative predictive value (NPV) were calculated at the optimal cut-off of the ROC curve, i.e., at the maximum Youden index. The joint diagnostic performance of CA 19-9 marker and miRNA levels (i.e., CA 19-9 + miRNA) was assessed using individual predicted probabilities (of being a PanC patient) derived from a multivariable logistic regression after including both variables as covariates. Improvement of the diagnostic performance achieved by the two biomarkers (i.e., CA 19-9 + miRNA) with respect to CA 19-9 alone was assessed by comparing the AUCs estimated from the two nested logistic models, following the De Long test (21).

Time-to-event analysis was performed in PanC patients only. Overall Survival (OS) was defined as the time between the diagnosis date and cancer-related death, whereas Disease-Free Survival (DFS) was defined as the time between diagnosis date and tumor progression. For patients who did not develop the event of interest, the endpoints (i.e., OS and DFS) were defined as the time between the diagnosis date and the last follow-up visit date. The association between miRNA plasma levels (as prognostic biomarker) and time-to-event outcomes were performed by univariable and multivariable proportional hazards Cox models. The latter included the candidate miRNA (categorized below and above the median cut-off) along with the following patients' covariates: age, preoperative tumor stage, tumor differentiation, AJCC tumor stage, and treatment. Risks were reported as hazard ratios (HR), along with their 95% CIs and adjusted survival curves were drawn with respect to the miRNA median cut-off.

A two-sided *p* < 0.05 was considered for statistical significance. All statistical analyses were performed using SAS Release 9.4 (SAS Institute, Cary, NC, USA), and plots were produced using R Foundation for Statistical Computing (version 3.6).

Raw microarray data were log-transformed and quantiles normalized. Probes mapping to any known miRNA surveyed in miRBase 22 were considered. In cases where several probesets mapped to the same miRNA, the one exhibiting the highest variance was chosen for further analysis. Batch effects were removed by Partek's batch effect removal algorithm. Expression similarity of samples within/between groups was assessed by means of the principal component analysis (PCA). Correction for multiple tests was achieved by the Benjamini–Hochberg procedure. Statistical differences in miRNA expression were assessed by the ANOVA test. The significance threshold was set to 0.05. MiRNA expression data were analyzed with Partek Genomics Suite 6.6. Data filtering and analyses were carried out with R Foundation for Statistical Computing (version 3.6).

Association of miRNAs to human diseases and biological functions was assessed using the BioProfiler tool of Ingenuity Pathway Analysis (IPA; QIAGEN, Redwood City, CA; www.qiagen.com/ingenuity), which yielded the effect of miRNAs on human diseases and biological functions, their expression evidences (up-, down-regulation), and whether a causal or correlation evidence existed between miRNAs and the reported diseases and functions in literature data.

## Results

### Discovery Phase

#### miRNA Expression Profiling in Pooled Sets of Plasma Samples

**Figure 2:**
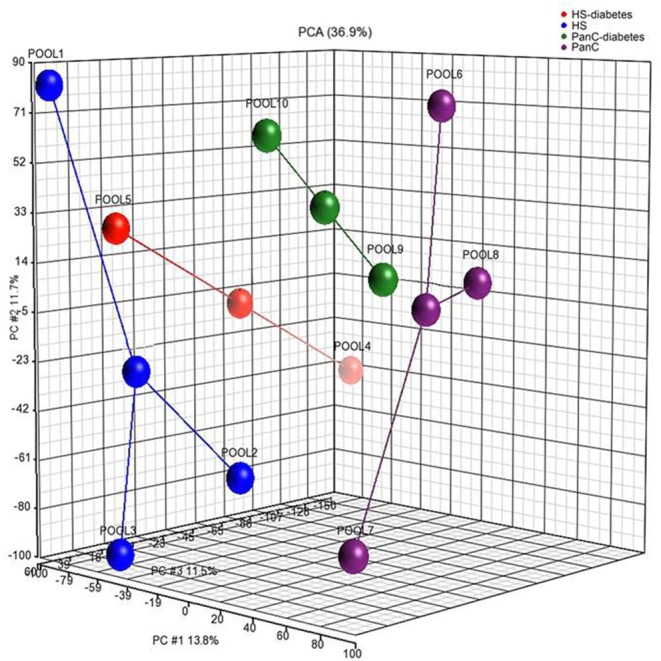
Principal component analysis performed on miRNAs expression levels in pooled plasma samples from healthy subjects and pancreatic cancer patients. Healthy subjects were stratified according to the category of risk for pancreatic cancer development (blue circles: Pool_1_, Pool_2_, Pool_3_), and to the date of diagnosis of diabetes (red circle: Pool_4_, Pool_5_); patients with pancreatic cancer were classified based on the preoperative stage of the disease (purple circles: Pool_6_, Pool_7_, Pool_8_), and on the date for patients diagnosis of diabetes (green circles: Pool_9_, Pool_10_).

Differences in miRNA expression levels between HS and PanC were evaluated by means of PCA. As shown in Figure 2, the pools from the HS cohort were spatially separated from those from PanC patients, implying a different expression of miRNAs between the two subsets. The PCA analysis also revealed close proximity between HS with early-onset diabetes (Pool_4_) and PanC. According to this spatial distribution, miRNA expression levels in pools from PanC patients (Pools _6−7−8−9−10_) were compared to those from HS (Pools _1−2−3−5_): at the *p* < 0.01 significance threshold, 82 miRNAs appeared differently expressed in PanC patients compared to HS subjects, with 51 up- and 31 down-regulated. The list of the 51 miRNAs over-expressed is shown in Table 2: the highest significant change (*p* < 0.0001) was evident for three miRNAs, namely miR-4666a-3p, miR-1273g-3p, and let-7b-5p.

**Table 2:** MicroRNA over-expressed in sets of pooled plasma from patients with pancreatic cancer compared to those from healthy controls.

	**Fold-change (95% CI)**	***P*-value**
miR-4666a-3p	1.28 (1.20–1.38)	<0.001
miR-1273g-3p	5.09 (3.08–8.42)	<0.001
let-7b-5p	1.64 (1.35–1.99)	<0.001
miR-122-5p	5.20 (2.68–10.11)	0.001
miR-6126	3.52 (2.09–5.93)	0.001
miR-1912	1.35 (1.19–1.53)	0.001
miR-3197	7.47 (3.20–17.45)	0,001
miR-4793-3p	7.91 (3.16–19.90)	0,001
miR-7107-5p	3.77 (2.08–6.85)	0.001
miR-1246	6.75 (2.67–17.10)	0.002
mir-4754	1.13 (1.06–1.20)	0.002
miR-4750-5p	1.25 (1.12–1.41)	0.002
miR-18a-5p	1.89 (1.36–2.62)	0.003
miR-619-5p	1.68 (1.28–2.21)	0.003
miR-4734	2.48 (1.54–4.00)	0.003
mir-6840	1.23 (1.10–1.38)	0.003
miR-1587	2.03 (1.40–2,96)	0.003
mir-487a	1.30 (1.13–1.51)	0.003
miR-4674	3.20 (1.70–6.04)	0.003
miR-193a-5p	5.17 (2.12–12.64)	0.003
miR-1275	2.13 (1.40–3.24)	0.004
miR-3195	1.79 (1.26–2.47)	0.004
miR-7108-5p	1.67 (1.25–2.22)	0.004
miR-548aq-5p	1.34 (1.14–1.59)	0.004
mir-4278	1.90 (1.04–1.14)	0.004
mir-2392	1.22 (1.09–1.38)	0.005
miR-324-3p	1.71 (1–34)	0.005
miR-7150	2.02 (1.34–3.04)	0.005
miR-4423-3p	1.23 (1.09–1.39)	0.005
miR-4717-3p	1.94 (1.31–2.87)	0.005
miR-4661-3p	1.26 (1.10–1.45)	0.005
miR-652-3p	2.96 (1.56–5.63)	0.005
miR-6855-3p	1.24 (1.09–1.41)	0.005
miR-1268b	1.72 (1.24–2.39)	0.006
miR-3152-5p	1.15 (1.06–1.25)	0.006
miR-6722-3p	1.82 (1.26–2.63)	0.006
mir-4674	1.26 (1.09–1.45)	0.007
miR-4486	2.40 (1.39–4.17)	0.007
miR-3162-5p	1.42 (1.14–1.77)	0.007
miR-4701-3p	1.91 (1.27–2.88)	0.007
miR-5093	1.88 (1.26–2.81)	0.007
miR-4507	2.20 (1.34–3.61)	0.007
miR-378a-3p	2.08 (1.31–3.30)	0.007
miR-223-3p	2.02 (1.29–3.15)	0.007
miR-5001-5p	1.77 (1.23-2.55)	0.007
miR-1301-3p	2.01 (1.29-3.14)	0.008
miR-4498	1.52 (1.15-1.99)	0.009
miR-4487	1.29 (1.09-1.53)	0.009
miR-4524b-3p	1.29 (1.09-1.53)	0.009
miR-6090	1.56 (1.16-2.10)	0.010
miR-3654	1.21 (1.06-1.38)	0.010

#### Selection of miRNAs for Further Validation Analyses

Since miRNAs with increased expression levels in cancer are considered to act as oncogenes in the development of the disease, miRNAs that may play an oncogenic role in the pancreatic carcinogenesis were selected from the 51 over-expressed miRNAs reported in Table 2 by combining two approaches. First, we selected the five miRNAs with the most significant increase in PanC compared to HS. Among these, miR-1273g-3p, miR-6126, and miR-122-5p showed also the highest fold-change values. In parallel, the 51 over-expressed miRNAs were prioritized by functional pertinence by means of the IPA BioProfiler tool, which was used to annotate the effect of miRNAs on human diseases and biological functions and whether a causal or correlation evidence existed between miRNAs and PanC in literature data. In particular, 12 miRNAs were selected because they were implicated in cancer-related biological functions (Supplementary Table 1); these miRNAs were further filtered according to: (i) their expression trends, namely only miRNAs that were previously reported as up-regulated were considered; by this approach the list of oncogenic miRNAs was reduced to 10; (ii) their effects on PanC; this condition restricted the number of miRNAs to two: miR-18a-5p and miR-223. After surveying pertinent literature on miR-223 in PanC, we uncovered for this miRNA a significant biological role in the processes of cell proliferation and disease progression, and we found different studies where miR-223 has been included in plasma miRNAs panels for the detection of PanC. Noteworthy, the same biological role in PanC resulted also for miR-122-5p, which was also individually tested in plasma from PanC patients and identified as an independent prognostic predictor (22). In addition, miR-122-5p exhibited a significantly higher fold-change expression value compared to miR-223 in our series, as mentioned above. Hence, supported by these considerations, we have selected miR-1273g-3p, miR-6126, miR-18a-5p, and miR-122-5p for further validation tests.

### Validation Phase

#### Absolute Quantification of Oncogenic miRNAs in Individual Plasma Samples

**Figure 3:**
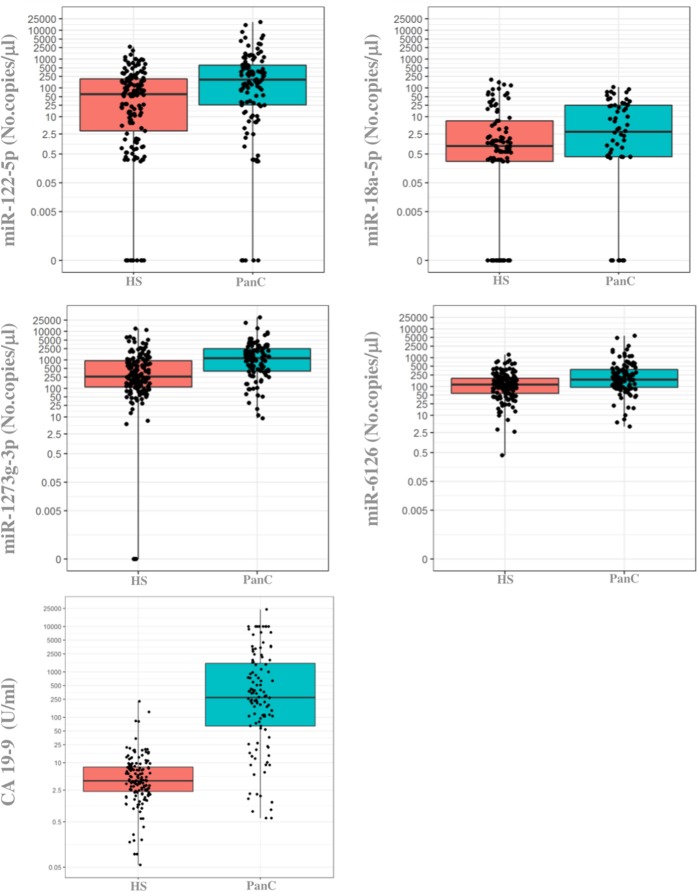
Boxplots of the absolute quantification of circulating levels of miRNAs and CA 19-9 in healthy subjects and in patients with pancreatic cancer. The ends of each box are the upper and lower quartiles, so the box spans the interquartile range (IQR), whereas the median is marked by a horizontal black line inside the box. The whiskers are the two lines outside the box that extend to the highest and lowest plotted observations (black dots). Values of both miRNAs (no.copies/μl) and CA 19-9 (U/ml) are reported in log scale (y-axis). PanC, pancreatic cancer; HS, Healthy subjects.

When the previously selected four miRNAs were assayed in the validation cohort of the PanC population and HS, three maintained a different expression between the two subsets when tested with the ddPCR: miR-122-5p, miR-1273g-3p, and miR-6126. As shown in Figure 3, the median circulating levels of these miRNAs were significantly higher in PanC patients than in HS subjects (*p* < 0.001): miR-122-5p [192.8 (25.8–618) vs. 60.0 (3.2–206.4)], miR-1273g-3p [1.158 (400–2.468) vs. 259.4 (110.4–948)] and miR-6126 [171.6 (92.8–390) vs. 115.2 (57.6–193.2)]. The median value for serum CA 19 9 (U/ml) were higher in PanC patients [273.15 (64.38–1537.5)] than in HS [3.98 (2.32–8.01)], *p* < 0.001 (Figure 2).

Median levels of miRNAs did not differ statistically in hemolyzed and no-hemolyzed samples, classified either according to the optical density of free hemoglobin at 414 nm, and to the lipemia-independent hemolysis score (Supplementary Table 2). Thus, all the samples were included in the following evaluations.

As reported in Supplementary Table 3, miRNAs plasma levels did not significantly differ between the low-, intermediate- and high-risk subgroups of PanC patients and HS. The CA 19 9 serum levels showed the same trend of association between the three subsets of subjects.

#### Diagnostic Performance of Oncogenic miRNAs Compared to the Conventional CA 19-9 Marker

**Figure 4:**
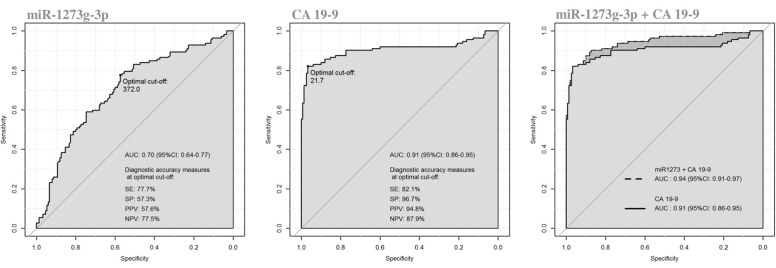
Receiver operating characteristics (ROC) curve analysis of miR-1273g-3p and CA 19-9 in discriminating patients with pancreatic cancer from healthy subjects.

We evaluated the diagnostic performance of the three significant oncogenic miRNAs, compared to CA 19-9 serum marker. As reported in Table 3, the diagnostic performance of serum CA 19-9 levels (AUC = 0.906, 95%CI: 0.861–0.952) was always higher than the ones achieved by each single miRNA, and each single miRNA marker achieved a less than acceptable discriminatory power (i.e., AUC Ca 0.70) (23). However, although the diagnostic performance achieved by the miR-1273g-3p alone was fairly good (AUC = 0.703, 95%CI: 0.639–0.768), the diagnostic performance achieved by both miR-1273g-3p and CA 19-9 levels (jointly considered) was significantly higher (AUC = 0.940, 95% CI: 0.909–0.972) than the one achieved by the serum CA 19-9 marker alone (*p* = 0.02 from DeLong test), Figure 4. Having classified subjects below or above the optimal cut-off found in the ROC curve of CA 19-9 + miR-1273g-3p (i.e., predicted probability of 0.189), very good operating characteristics were detected when assuming that subjects below the cut-off were considered as being HS and those above the cut-off were considered as being PanC patients: SE = 90.2%, SP = 87.3%, PPV = 84.2% and NPV = 92.3%, although only SE and NPV were higher than those found on the two single markers (for CA 19-9: SE = 82.1% and NPV = 87.9%; for miR-1273g-3p: SE = 77.7% and NPV = 77.5%).

**Table 3:** Diagnostic performance achieved by a receiver operating characteristic (ROC) curve analysis for miR-1273g-3p, miR-122-5p and miR-6126 compared to serum CA 19-9 determination in distinguishing patients with pancreatic cancer from healthy subjects.

		**CA 19-9**	**miRNA**	**CA 19-9 + miRNA**	*p*-value^*^
**miR-1273g-3p**	AUC (95%CI)	0.906 (0.861–0.952)	0.703 (0.639–0.768)	0.940 (0.909–0.972)	**0.020**
	Optimal cutoff value	21.71	372	0.189	
	SE (%)	82.1%	77.7%	90.2%	
	SP (%)	96.7%	57.3%	87.3%	
	PPV (%)	94.8%	57.6%	84.2%	
	NPV (%)	87.8%	77.5%	92.3%	
**miR-122-5p**	AUC (95%CI)		0.658 (0.590–0.726)	0.933 (0.899–0.967)	0.086
	Optimal cutoff value		243.6	0.272	
	SE (%)		49.1%	83.9%	
	SP (%)		79.2%	94.6%	
	PPV (%)		64.0%	92.2%	
	NPV (%)		67.4%	88.7%	
**miR-6126**	AUC (95%CI)		0.644 (0.576–0.712)	0.917 (0.877–0.956)	0.362
	optimal cutoff value		167.2	0.290	
	SE (%)		54.5%	83.9%	
	SP (%)		67.8%	93.3%	
	PPV (%)		56%	90.4%	
	NPV (%)		66.4%	88.5%	
**miR-1273g-3p+** **miR-122-5p+** **miR-6126**	AUC (95%CI)		0.716 (0.652–0.779)	0.936 (0.904–0.968)	0.058
	Optimal cutoff value		0.466^∧^	0.435^∧^	
	SE (%)		64.3%	82.1%	
	SP (%)		70.9%	95.3%	
	PPV (%)		62.6%	92.9%	
	NPV (%)		72.4%	87.6%	

*CI, confidence interval; SE, sensibility; SP, specificity, PPV, positive predictive value; NPV, negative predictive value*.

*SE, SP, PPV, and NPV measures were calculated at the optimal cut-offs*.

* *Comparisons between two AUCs estimated using the individual predicted probabilities (of being a PanC patient) derived from CA 19-9 + miRNA and CA 19-9 nested logistic models (p-value from DeLong test). Value in bold indicates a statistically significant difference*.

*The optimal cut-off in CA 19-9 + miRNA model was referred to such individual predicted probability*.

∧ *optimal cut-off referred to predicted probabilities from logistic models*.

Moreover, we determined the diagnostic performance of the combination between all the three oncogenic miRNAs (i.e., miR-1273g-3p, miR-122-5p and miR-6126). As reported in Table 3, although a less than acceptable discriminatory power persisted for this miRNAs panel, the combination of the three miRNAs with the CA 19-9 determinations (jointly considered) showed a trend toward a better discriminatory ability (AUC = 0.936; 95%CI: 0.904-0.968) compared to the CA 19-9 test alone (*p* = 0.058 from DeLong test).

#### Levels of Circulating miRNAs in Non-Cancer Internal Controls

To ascertain the ability of significant oncogenic miRNAs to discriminate non-malignant pancreatic diseases from HS and PanC patients, miRNAs expression levels were also sought in two small subsets of internal controls consisting of patients with IPMN and chronic pancreatitis. Data are shown in Supplementary Table 4. Plasma levels of miR-122-5p, miR-1273g-3p, and miR-6126 were not statistically different between IPMN and chronic pancreatitis and were not able to differentiate non-malignant controls from PanC patients. In addition, median levels of miR-6126 did not statistically differ between non-cancer internal controls and HS. Conversely, the expression of miR-122-5p and miR-1273g-3p was significantly increased in patients with IPMN and chronic pancreatitis compared to HS. The CA 19-9 tumor marker showed the potential to discriminate non-malignant subjects from either HS and PanC: patients with non-malignant pancreatic disease had higher CA19-9 serum levels compared to HS, while the median values of the tumor marker were lower in plasma from IPMN and chronic pancreatitis than in those from PanC.

#### miRNA Plasma Levels in Association With Clinical Phenotypes and Outcomes

**Figure 5:**
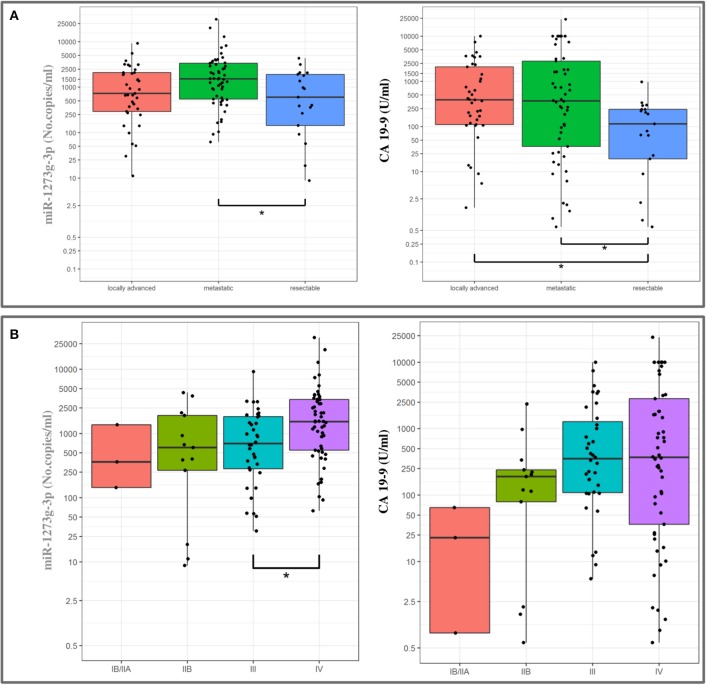
Association between miR-1273g-3p plasma levels and CA 19-9 values with pre-operative classification **(A)** and tumor stage **(B)** in patients with pancreatic cancer (boxplots). The ends of each box are the upper and lower quartiles, so the box spans the interquartile range (IQR), whereas the median is marked by a horizontal black line inside the box. The whiskers are the two lines outside the box that extend to the highest and lowest plotted observations (black dots). Values are reported in log scale (y-axis).

High expression levels were associated with a poor prognosis for all the miRNAs (Table 4), except for miR-18a-5p (data not shown). In detail, circulating miR-1273g-3p levels were directly correlated with the AJCC tumor stage and with the preoperative stage of the disease, as median expression values were increased in patients with a metastatic or a locally spread disease (Figure 5). As shown in Figure 5, a similar pattern appeared for the CA 19-9 serum levels. In addition, miR-122-5p and miR-6126 levels were higher in metastatic PanC patients compared to those without a metastatic cancer, and plasma levels of miR-122-5p were significantly associated with pT-stage.

**Table 4:** Associations of clinical-pathological features associated with oncogenic miRNAs and CA 19-9 levels in patients with pancreatic cancer.

	**miR-122-5p**			**miR-1273g-3p**			**miR-6126**			**CA 19-9**		
	**No**	**Median (IQR)**	***p*-value**	**No**	**Median (IQR)**	***p*-value**	**No**	**Median (IQR)**	***p*-value**	**No**	**Median (IQR)**	***p*-value**
**Pre-operative classification**												
Resectable	19	165 (2.44–360)	0.160	19	604 (144–1,908)	**0.019**	19	167 (84.8–222)	0.078	19	114 (19.3–240)	**0.017**
Locally advanced	39	118 (23.2–580)		39	732 (292–2092)		39	159 (83.2–324)		39	389 (111–2109)	
Metastatic	54	269 (68.8–1,072)		54	1,528 (548–3376)		54	221 (116–632)		54	369 (36.3–2,826)	
**Tumor size: pT**												
2	4	50.0 (0.16–216)	**0.031**	4	252 (77.4–380)	**<0.0001**	4	127 (47.4–397)	0.085	4	89.6 (43.8–1,230)	0.164
3	16	151 (1.76–604)		16	654 (277–2,002)		16	204 (98.4–242)		16	154 (19.2–288)	
4	60	142 (24.0–618)		60	894 (313–1,974)		60	155 (84.8–326)		60	366 (106–1,862)	
X	26	366 (138–1,796)		26	2274 (1,260–4,056)		26	406 (118–1,108)		26	431 (21.7–1,821)	
**Distant Lymph nodes: pN**												
0	52	128 (6.60–536)	**0.054**	52	666 (269–1,844)	**0.002**	52	158 (82.2–279)	**0.032**	52	220 (92.2–861)	0.285
1	54	269 (68.8–1,072)		54	1,528 (548–3,376)		54	221 (116–632)		54	369 (36.3–2,826)	
**Tumor stage**												
IB/IIA	3	99.6 (0.00–1,316)	0.222	3	360 (144–1,368)	**0.015**	3	170 (84.8–235)	0.168	3	22.8 (0.84–64.9)	**0.057**
IIB	13	138 (0.92–360)		13	604 (266–1,908)		13	159 (74.8–222)		13	190 (79.4–240)	
III	36	146 (24.0–788)		36	700 (282–1,844)		36	153 (82.2–326)		36	353 (109–1,286)	
IV	54	269 (68.8–1,072)		54	1,528 (548–3,376)		54	221 (116–632)		54	369 (36.3–2,826)	
**Adjuvant therapy**												
No	46	269 (32–1,140)	0.228	46	1,316 (548–3,152)	0.079	46	214 (125–444)	0.123	46	284 (25.5–2,109)	0.827
Yes	64	166 (19.8–498)		64	1000 (290–2,088)		64	159 (84.8–313)		64	284 (76.5–1,018)	

*IQR, interquartile range (i.e., first-third quartiles). Values in bold indicate a statistically significant difference*.

**Figure 6:**
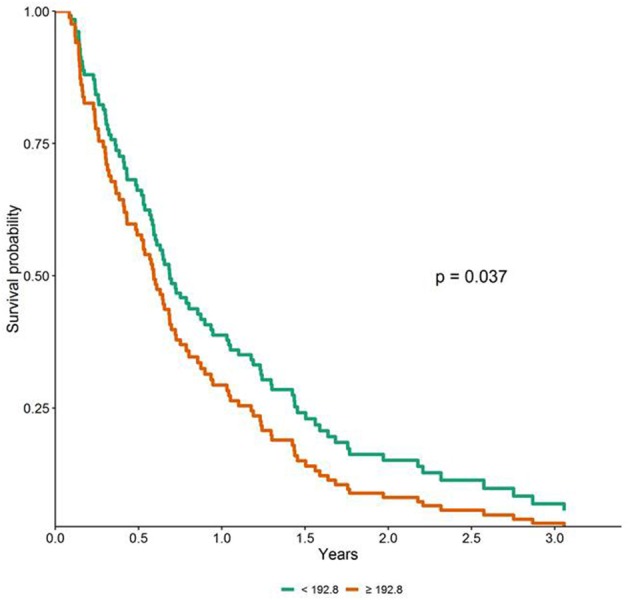
Adjusted survival curves for overall survival (OS) in patients with pancreatic cancer according to miR-122-5p plasma levels (i.e., below and above the median value).

To perform the survival analysis, PanC patients were sorted out, according to the cut-off value of the miRNA expression levels, in subjects with low or high concentrations. Univariable Cox analyses uncovered a significant worst OS in patients with miR-122-5p levels above the median cut-off value (HR = 1.73, 95% CI = 1.16–2.59, *p* = 0.008), whereas no significant association emerged for miR-1273g-3p, miR-18a-5p and miR-6126. As for DFS, no significant difference emerged between patients with low or high concentrations for all the tested miRNAs. As reported in Supplementary Table 5, the relationship between age, gender, pathological features and treatment with OS/DFS were also assessed. To downplay the effect of selection bias, which could affect the true associations between miRNA plasma levels and patients clinical phenotypes and outcomes, multivariable Cox analysis was performed and, as shown in the survival plot (Figure 6), high miR-122-5p plasma levels persisted as independently associated only with worst OS in PanC patients (HR = 1.58, 95% CI = 1.03–2.43, *p* = 0.037).

## Discussion

Early detection of PanC remains a great challenge for either clinicians and researchers. Current screening techniques foresee invasive and expensive tests, and the conventional CA 19-9 marker is not reliable in diagnosing the PanC at an early stage of development (24). Scientists are still searching for new specific, sensible and cost-effective biomarkers in the attempt to implement a screening protocol based on a combination of biomarkers and imaging of the pancreas (22–26). Herein we attempted to uncover circulating miRNAs that would enhance the power of serum CA 19-9 determination in identifying PanC.

In the present investigation, bioinformatics selection of differentially expressed miRNAs in plasma uncovered miR-18a-5p, miR-122-5p, miR-1273g-3p, and miR-6126 as candidate oncogenic biomarkers for the identification of PanC patients. By using the ddPCR technology we were able to validate the significant over-expression of miR-122-5p, miR-1273g-3p, and miR-6126 in PanC compared to HS, in line with the trend of the CA 19-9 levels. Conversely, no significant difference in miR-18a-5p levels between cancer patients and HS emerged in our study population. Accordingly, the literature reported controversial data about this miRNA in PanC: although higher circulating miR-18a-5p levels in patients with PanC compared to healthy controls have been reported, the level of this miRNA was found to be not significantly increased in the serum of human KRAS oncogene transgenic rats with ductal PanC (11, 27).

Next, we evaluated the diagnostic performance of each of the three significant miRNAs as well as that of the serum CA 19-9 evaluation: as shown in Table 3, the diagnostic accuracy of the latter biomarker was superior to that associated with the evaluation of each of the three miRNAs. However, we found that miR-1273g-3p (and almost the miR-122-5p) achieved a significant synergistic effect in enhancing the outstanding diagnostic accuracy of CA 19-9 (AUC > 0.90). Indeed, it is widely known (28–30) that once the AUC reaches a certain level, it usually requires unrealistically large effect sizes from new variables to lead to any noticeable increase, but it was not the case, because both the miRNAs would carry important additional information (needed for distinguish patients from healthy subjects) that the only CA 19-9 did not take into account. In detail, the diagnostic power of the combination of miRNA-1273g-3p with the co-determination of serum CA 19-9 proved to have a specificity value similar to that of the single assay of CA 19-9, but a better scoring for the sensitivity value and the negative predictive value. As a consequence, the AUC value achieved by the contemporary evaluation of the miR-1273g-3p and CA 19-9 was significantly higher than the value registered for the two biomarkers tested alone. On clinical grounds, it was apparent that the combined evaluation of the miR-1273g-3p and the CA 19-9 resulted in a lower false-negative rate with respect to the determination of the last biomarker alone. Few patients with PanC would escape the right ascertainment of the malignancy by the simultaneous determination of the miRNA and CA 19-9. If a screening program would have to be implemented for the detection of PanC, assay(s) with high sensibility power would help identify patients in need of deeper evaluation, while assay(s) with high specificity power would leave out from further examination many patients who will harbor a PanC.

It is worth mentioning that in our cohorts of patients with inflammatory or proliferative, but not neoplastic, conditions of the pancreatic parenchyma, i.e., in those with a chronic pancreatitis or an IPMN, median values of miRNAs in plasma were not statistically different from those observed in PanC patients, a likely explanation for the low specificity value registered with these biomarkers. These results underline the notion that none of the oncogenic miRNAs had the potential to distinguished between a neoplastic and a proliferative/inflammatory disease of the pancreas.

As a further investigative step, we analyzed whether plasma values of the three selected miRNAs could be related to the presence of risk factors for PanC development. We did the analysis by stratifying either cancer patients and HS according to the number of risk factors at their presentation. Either in cancer patients and HS, median plasma levels of miRNAs did not vary according to the number of risk factors: patients with the highest number of risk factors (i.e., 4 or more) presented with similar levels as those with a lower number. A similar pattern was also appreciated among the HS subset, implying that the evaluated miRNAs could not serve as biomarkers to be implemented in a screening or surveillance program. The only valuable association in PanC patients was found between miR-1273g-3p and tumor stage, and between miR-122 and a poor overall survival: high levels of miR-1273g-3p were associated with more advanced tumor stage and increased miR-122-5p expression in plasma was identified as independent negative prognostic factor for patients with PanC. These data suggested a role for these miRNAs in predicting the clinical outcomes of PanC patients.

In keeping with our findings, different studies identified the up-regulation of miR-122-5p in plasma or whole blood from patients with PanC (15, 22, 31). However, controversial results have been reported about the alteration of this miRNA in matched-pairs of normal and tumoral PanC tissue specimens. Therefore, even if the up-regulated levels of miR-122-5p in plasma might be associated with some specific response of patients with PanC, the role of miR-122-5p in PanC has been reported to require further investigations. Herein we provided the first evidence about the association between the increase of miR-122-5p plasma levels and the worst prognosis of patients with PanC.

As for miR-6126 and miR-1273g-3p, our findings provided the first evidence of alterations of these miRNAs in plasma from PanC patients. To date, the deregulation of miR-6126 in PanC has not yet been described, and the only evidence on miR-6216 deregulation in cancer is about the over-expression of miR-6126 in tissues from colon cancer patients compared to controls, and in healthy ovarian tissue compared to ovarian cancer samples where it acts as a tumor suppressor via integrin β1 (32, 33). Similarly, few data are available on miR-1273g-3p in PanC patients. Rachagani and colleagues, by using the KrasG12D; Pdx1-Cre mouse model to investigate the global miRNA expression profile during the PanC progression found increased levels of miR-1273g-3p in mice aged from 10 to 50 weeks, and corroborated this alteration in human PanC cell lines and tissues (34). The up-regulation of miR-1273g-3p and its involvement in promoting cell migration, proliferation, and invasion via CNR1 gene have also been described in both lung and colorectal cancer cell lines (35, 36). Furthermore, several binding sites for miR-1273g-3p have been identified on mRNAs of several other genes (NOL9, PLCXD1, ZNF490, CYP20A1, GNL3L, PPM1K, RBMS2, SAR1B, SLC35E2, IRCQ, ZNF850, MDM4) by using the target gene prediction program (37).

In conclusion, the present investigation was able to highlight three miRNAs, miR-1273g-3p, miR-122-5p, and miR-6126, differentially expressed in plasma samples from PanC patients and HS. None of these miRNAs exhibited the potential to distinguish between a neoplastic and a proliferative/inflammatory disease of the pancreas, and as to the implementation of a screening program by adopting the plasma assay of miRNAs, these biomarkers did not help identify individuals at high risk for PanC development. On the clinical relevance, the diagnostic performance of assaying the miR-1273g-3p in combination with serum CA 19-9 levels allowed a higher number of patients who will eventually be diagnosed with a PanC at further examination. In addition, miR-1273g-3p and miR-122-5p plasma levels were associated with worse prognosis and clinical outcome. Overall, although further investigations are needed to validate the biological mechanism of the identified miRNAs in PanC, our study highlighted new diagnostic and prognostic circulating miRNAs in PanC.

## Data Availability Statement

The datasets generated for this study have been deposited in ArrayExpress (accession number E-MTAB-8378).

## Ethics Statement

The studies involving human participants were reviewed and approved by Ethics Committee, Fondazione IRCCS Casa Sollievo della Sofferenza, San Giovanni Rotondo, Foggia, Italy. The patients/participants provided their written informed consent to participate in this study.

## Author Contributions

FT conceived the study and wrote the manuscript. DG performed the experiments. TM and TB performed the bioinformatics analyses. MC and OP performed the microarray experiments. AF performed the statistical analysis. EM and FB contributed to the collection of the biological sample. AA critically revised the manuscript. All the authors reviewed and approved the final draft.

### Conflict of Interest

The authors declare that the research was conducted in the absence of any commercial or financial relationships that could be construed as a potential conflict of interest.

## Supplementary Material

Supplementary Table 1 BioProfiler analysis for selection of oncogenic miRNAs in pancreatic cancer.

Supplementary Table 2 MiRNAs levels in plasma with low grade hemolysisi compared to not hemolysed plasma samples. Values are reported as medians along with interquartile range.

Supplementary Table 3 Associations between clinical risk factors for pancreatic cancer (PanC) development and circulating levels of candidate oncogenic miRNAs and CA 19–9.

Supplementary Table 4 Absolute quantification of circulating oncogenic miRNAs and CA 19–9 in subjects with intraductal papillary mucinous neoplasm (IPMN) of the pancreas and chronic pancreatitis (CP) compared with healthy subjects (HS) and pancreatic cancer (PanC) patients. Values are reported as medians along with interquartile range.

Supplementary Table 5 Univariable Cox analyses evaluating the association between miRNAs plasma levels (and clinical parameters) with overall survival (OS) and disease free survival (DFS) of patients with pancreatic cancer.

## References

[1] Siegel R Naishadham D Jemal A. Cancer statistics, 2013. CA Cancer J Clin. (2013) 63:11–30. 10.3322/caac.21166 23335087

[2] Bartsch DK Gress TM Langer P. Familial pancreatic cancer—current knowledge. Nat Rev Gastroenterol Hepatol. (2012) 9 445–53. 10.1038/nrgastro.2012.111 22664588

[3] Becker AE Hernandez YG Frucht H Lucas AL. Pancreatic ductal adenocarcinoma: risk factors, screening, and early detection. World J Gastroenterol. (2014) 20:11182–98. 10.3748/wjg.v20.i32.11182 25170203 PMC4145757

[4] Wolpin BM Chan AT Hartge P Chanock SJ Kraft P Hunter DJ. ABO blood group and the risk of pancreatic cancer. J Natl Cancer Inst. (2009) 101:424–31. 10.1093/jnci/djp020 19276450 PMC2657095

[5] Pannala R Basu A Petersen GM Chari ST. New-onset diabetes: a potential clue to the early diagnosis of pancreatic cancer. Lancet Oncol. (2009) 10:88–95. 10.1016/S1470-2045(08)70337-1 19111249 PMC2795483

[6] Gupta S Vittinghoff E Bertenthal D Corley D Shen H Walter LC. New-onset diabetes and pancreatic cancer. Clin Gastroenterol Hepatol. (2006) 4:1366–72. 10.1016/j.cgh.2006.06.024 16945591

[7] Gilad S Meiri E Yogev Y Benjamin S Lebanony D Yerushalmi N. Serum microRNAs are promising novel biomarkers. PLoS ONE. (2008) 3:e3148. 10.1371/journal.pone.0003148 18773077 PMC2519789

[8] Slotwinski R Lech G Słotwinska SM. MicroRNAs in pancreatic cancer diagnosis and therapy. Cent Eur J Immunol. (2018) 43:314–24. 10.5114/ceji.2018.80051 30588176 PMC6305615

[9] Abue M Yokoyama M Shibuya R Tamai K Yamaguchi K Sato I. Circulating miR-483-3p and miR-21 is highly expressed in plasma of pancreatic cancer. Int J Oncol. (2015) 46:539–47. 10.3892/ijo.2014.2743 25384963 PMC4277249

[10] Morimura R Komatsu S Ichikawa D Takeshita H Tsujiura M Nagata H. Novel diagnostic value of circulating miR-18a in plasma of patients with pancreatic cancer. Br J Cancer. (2011) 105:1733–40. 10.1038/bjc.2011.453 22045190 PMC3242609

[11] Komatsu S Ichikawa D Takeshita H Morimura R Hirajima S Tsujiura M. Circulating miR-18a: a sensitive cancer screening biomarker in human cancer. In Vivo. (2014) 28:293–7. 24815829

[12] Liu R Chen X Du Y Yao W Shen L Wang C. Serum microRNA expression profile as a biomarker in the diagnosis and prognosis of pancreatic cancer. Clin Chem. (2012) 58:610–8. 10.1373/clinchem.2011.172767 22194634

[13] Kawaguchi T Komatsu S Ichikawa D Morimura R Tsujiura M Konishi H. Clinical impact of circulating miR-221 in plasma of patients with pancreatic cancer. Br J Cancer. (2013) 108:361–9. 10.1038/bjc.2012.546 23329235 PMC3566805

[14] Liu J Gao J Du Y Li Z Ren Y Gu J. Combination of plasma microRNAs with serum CA19-9 for early detection of pancreatic cancer. Int J Cancer. (2012) 131:683–91. 10.1002/ijc.26422 21913185

[15] Schultz NA Dehlendorff C Jensen BV Bjerregaard JK Nielsen KR Bojesen SE. MicroRNA biomarkers in whole blood for detection of pancreatic cancer. JAMA. (2014) 311:392–404. 10.1001/jama.2013.284664 24449318

[16] Tavano F Gioffreda D Valvano M.R Palmieri O Tardio M Latiano TP. Droplet digital PCR quantification of miR-1290 as a circulating biomarker for pancreatic cancer. Sci Rep. (2018) 8:16389. 10.1038/s41598-018-34597-z 30401891 PMC6219528

[17] Li A Yu J Kim H Wolfgang CL Canto MI Hruban HR. MicroRNA array analysis finds elevated serum miR-1290 accurately distinguishes patients with low-stage pancreatic cancer from healthy and disease controls. Clin Cancer Res. (2013) 19:3600–10. 10.1158/1078-0432.CCR-12-3092 23697990 PMC3707520

[18] Xie K Wolff R Abbruzzese JL. Pancreatic cancer. Lancet. (2004) 363:1049–57. 10.1016/S0140-6736(04)15841-8 15051286

[19] Edge SB Compton CC (editors) Exocrine and endocrine pancreas. In: AJCC Cancer Staging Manual 7th Edn New York, NY: Springer (2010) p. 241–9. 10.1007/978-0-387-88441-7_24

[20] Moret I Sánchez-Izquierdo D Iborra M Tortosa L Navarro-Puche A Nos P. Assessing an improved protocol for plasma microRNA extraction. PLoS ONE. (2013) 8:e82753. 10.1371/journal.pone.0082753 24376572 PMC3871541

[21] DeLong ER DeLong DM Clarke-Pearson DL. Comparing the areas under two or more correlated receiver operating characteristic curves: a nonparametric approach. Biometrics. (1988) 44:837–45. 10.2307/2531595 3203132

[22] Zhou X Lu Z Wang T Huang Z Zhu W Miao Y. Plasma miRNAs in diagnosis and prognosis of pancreatic cancer: a miRNA expression analysis. Gene. (2018) 673:181–93. 10.1016/j.gene.2018.06.037 29913239

[23] Hosmer DW Lemeshow S (2000). Applied Logistic Regression. 2nd ed New York, NY: John Wiley & Sons, Inc.

[24] Chari ST Kelly K Hollingsworth MA Thayer SP Ahlquist DA Andersen DK. Early detection of sporadic pancreatic cancer: summative review. Pancreas. (2015) 44:693–712. 10.1097/MPA.0000000000000368 25931254 PMC4467589

[25] Ghatnekar O Andersson R Svensson M Persson U Ringdahl U Zeilon P. Modelling the benefits of early diagnosis of pancreatic cancer using a biomarker signature. Int J Cancer. (2013) 133:2392–7. 10.1002/ijc.28256 23649606

[26] Kenner BJ Chari ST Cleeter DF Go VL Early detection of sporadic pancreatic cancer: strategic map for innovation-a white paper. Pancreas. (2015) 44:686–92. 10.1097/MPA.0000000000000369 25938853 PMC4467583

[27] Yabushita S Fukamachi K Tanaka H Sumida K Deguchi Y Sukata T. Circulating MicroRNAs in serum of human K-ras oncogene transgenic rats with pancreatic ductal adenocarcinomas. Pancreas. (2012) 41:1013–8. 10.1097/MPA.0b013e31824ac3a5 22513294

[28] Ware JH. The limitations of risk factors as prognostic tools. N Eng J Med. (2006) 355 2615–7. 10.1056/NEJMp068249 17182986

[29] Cook NR Use and misuse of the receiver operating characteristics curve in risk prediction. Circulation. (2007) 115:928–35. 10.1161/CIRCULATIONAHA.106.672402 17309939

[30] Pepe MS Janes H Longton G Leisenring W Newcomb P Limitations of the odds ratio in gauging the performance of a diagnostic, prognostic, or screening marker. Am J Epidemiol. (2004) 159:882–90. 10.1093/aje/kwh101 15105181

[31] Bauer AS Keller A Costello E Greenhalf W Bier M Borries A. Diagnosis of pancreatic ductal adenocarcinoma and chronic pancreatitis by measurement of microRNA abundance in blood and tissue. PLoS ONE. (2012) 7:e3415. 10.1371/journal.pone.0034151 22511932 PMC3325244

[32] Gungormez C Gumushan Aktas H Dilsiz N Borazan E. Novel miRNAs as potential biomarkers in stage II colon cancer: microarray analysis. Mol Biol Rep. (2019) 46:4175–83. 10.1007/s11033-019-04868-7 31123908

[33] Kanlikilicer P Rashed MH Bayraktar R Mitra R Ivan C Aslan B. Ubiquitous release of exosomal tumor suppressor miR-6126 from ovarian cancer cells. Cancer Res. (2016) 76:7194–207. 10.1158/0008-5472.CAN-16-0714 27742688 PMC5901763

[34] Rachagani S Macha MA Menning MS Dey P Pai P Smith LM. Changes in microRNA (miRNA) expression during pancreatic cancer development and progression in a genetically engineered KrasG12D;Pdx1-Cre mouse (KC) model. Oncotarget. (2015) 6:40295–309. 10.18632/oncotarget.5641 26516699 PMC4741896

[35] Hou L Su X Qing X Ding H Huang A Li H MiR-1273g-3p regulates A549 cell migration by targeting CNR1. J Med Mol Biol. (2014) 11 125–31. 10.1002/1873-3468.12309

[36] Li M Qian X Zhu M Li A Fang M Zhu Y miR-1273g-3p promotes proliferation, migration and invasion of LoVo cells via cannabinoid receptor 1 through activation of ERBB4/PIK3R3/mTOR/S6K2 signaling pathway. Mol Med Rep. (2018) 17:4619–26. 10.3892/mmr.2018.8397 29328379

[37] Ivashchenko A Berillo O Pyrkova A Niyazova R Binding sites of miR-1273g-3p family on the mRNA of target genes. Biomed Res Int. (2014) 2014:620530 10.1155/2014/620530 25243165 PMC4160624

